# The Treatment of Wounds Controversy

**Published:** 1915-10-23

**Authors:** 


### The Hospital

The Workers' Newspaper of Administrative Medicine and Institutional Life, Administration, National Insurance and Health.

No. 1532, Vol. LIX. SATURDAY, OCTOBER 23, 1915. PRICE ONE PENNY.

### THE TREATMENT OF WOUNDS CONTROVERSY.

The controversy which has arisen in reference to the treatment of wounds has at least one redeeming feature—it is concerned not with petty details, but with large principles. At first sight it must seem strange, at least to the non-medical person, that a difference about such an apparently elementary matter can exist at all. Wounds and surgical dealings with them go back to the beginnings of things almost, and yet here we are to-day disputing of their reciprocal relations. The experience is an impressive illustration of the complexity of many things which on the surface seem simple, and of the enormous difficulty of disentangling truth from error in human affairs. A wound and the healing of a wound do not seem to present any difficult problem until the necessity of reviewing the whole subject is forced on us by the multiplied casualties of a great war. Then it appears that everything is once more in the melting-pot, and no one can say at the moment in what shape opinion will finally crystallise itself.

The story commences with Lord Lister's teaching that many of the local complications which follow wounds, and still more the general sepsis or blood-poisoning then so frequently seen in hospital wards, were due to the introduction of microbes. Hence his practice to keep out the microbes from the wounds made by the surgeon and to kill them by antiseptics in any wound to which they had already gained access. The record can be summed up in a sentence, though years of work and of struggle against opposition were needed to secure the reading of it. Next came the suggestion that surgeons might inflict wounds with safety provided that the operator, his assistants, and the instruments were microbe free, so far at least as contact with the wound was concerned; and that this condition could be satisfied without the use of chemical agents or antiseptics. Here was the school of aseptic surgery claimed by some to be a negation of Lord Lister's system; but in reality being only a different method of securing the end he taught us to desire—namely, the protection of wounds from microbic infection. Manifestly this aseptic method was not available for wounds already infected, as in the common accidents of daily life, so that here the application of antiseptics continued to be practised. Now comes the war with its tens of thousands of wounds, and the enormous majority septic either from the moment of their infliction or very shortly afterwards. Inevitably the call for antiseptic applications became predominant, and the only question was which agent was the best and which the best method of procedure. Different surgeons held different views on these two points, and naturally each favoured the plan to which he was accustomed. But in all this there was no serious divergence on a point of principle. The aim of all alike was to kill the microbes and to prevent their multiplication in the wound.

The latest development is a challenge of the trustworthiness of any antiseptic applications. The proposition is that these agents cannot be relied on to kill the microbic invaders of wounds, or at least not to kill all of them; and a partial destruction is soon nullified by the rapid multiplication which marks the career of these lowly forms of life. On the other hand, it is claimed that by the continual flow of certain saline solutions over the exposed surface of a wound there are promoted tissue operations which are successful against the invading organisms; and evidence both from the laboratory and from the hospital ward is produced in support of these propositions. It is here that the battle is joined. As was to be expected, many surgeons with a long experience of the value of antiseptics could only view with dismay a proposal to abandon methods which had long served them well. When they are told that the wound infections of civil life are comparatively trifling, and that what may suffice for these is unequal to the more virulent infections of the battlefield, they deny the proposition and contend that all that is wanted is a modification in detail to meet the new position. Some of them devise methods to secure this modification, and, when unsatisfactory results are reported, explain the failures as due to imperfect application; and they give reasons for this conclusion. This is the point at which we now stand, and only experience will decide the issue. What is of most general interest on the situation is, perhaps, the exhibition of the readiness with which the profession listens to evidence in favour of what is nothing less than a revolutionary change in surgical practice.

